# Validation of the 2018 FIGO Classification for Cervical Cancer: Lymphovascular Space Invasion Should Be Considered in IB1 Stage

**DOI:** 10.3390/cancers12123554

**Published:** 2020-11-28

**Authors:** Vincent Balaya, Benedetta Guani, Laurent Magaud, Hélène Bonsang-Kitzis, Charlotte Ngô, Patrice Mathevet, Fabrice Lécuru, on behalf of the SENTICOL Group

**Affiliations:** 1 Gynecology Department, Centre hospitalo-universitaire vaudois, 1011 Lausanne, Switzerland; Benedetta.Guani@chuv.ch (B.G.); Patrice.Mathevet@chuv.ch (P.M.); 2 Faculty of Biology and Medicine, University of Lausanne, 1015 Lausanne, Switzerland; 3 Hospices Civils de Lyon, Pôle Santé Publique, Service recherche et épidémiologie cliniques, F-69003 Lyon, France; laurent.magaud@chu-lyon.fr; 4 Université Claude Bernard Lyon 1, HESPER EA 7425, F-69008 Lyon, France; 5 Gynecological and Breast Surgery and Cancerology Center, RAMSAY-Générale de Santé, Hôpital Privé des Peupliers, 75013 Paris, France; h.kitzisbonsang@yahoo.fr (H.B.-K.); charlotte.ngo2@gmail.com (C.N.); 6 Breast, Gynecology and Reconstructive Surgery Department, Curie Institute, 75006 Paris, France; fabrice.lecuru@curie.fr

**Keywords:** cervical cancer, FIGO classification, Lymphovascular space invasion, oncologic outcomes, SENTICOL, disease-free survival

## Abstract

**Simple Summary:**

The purpose of modifying a tumor staging system is to incorporate already well-established prognostic factors, allowing one to stratify cases and leading to tailored treatment approaches. Although lymphovascular space invasion (LVSI) has been described as an independent risk-factor of recurrence in early-stage cervical cancer and defined intermediate and high-risk cervical cancer according to the ESGO (European Society of Gynaecological Oncology) guidelines, this factor remains controversial and was not included in the last revised 2018 International Federation of Gynecology and Obstetrics (FIGO) classification. The aim of the present study was to determine whether LVSI has an impact on the prognosis of IB1 patients according to 2018 FIGO classification through two French prospective multicentric cohorts. Our results highlighted that LVSI was associated with a significantly decreased 5-year DFS in IB1 2018 FIGO stage compared to negative LVSI. Particular attention should be paid to LVSI status in early-stage cervical cancer for a more precise risk assessment and we suggest that LVSI may be considered in the new 2018 FIGO classification.

**Abstract:**

Background: The aim of this study was to assess the prognostic impact of Lymphovascular space invasion (LVSI) in IB1 stage of the revised 2018 International Federation of Gynecology and Obstetrics (FIGO) classification for cervical cancer. Methods: A secondary analysis of two French prospective multicentric trials on Sentinel Lymph node biopsy for cervical cancer was performed. Patients with 2009 FIGO IB1 stage who underwent radical surgery between January 2005 and July 2012 from 28 French expert centers were included. The stage was modified retrospectively according to the new 2018 FIGO staging system. Results: According to the 2009 FIGO classification, 246 patients had IB1 disease stage and fulfilled the inclusion criteria. The median follow-up was 48 months (4–127). Twenty patients (8.1%) experienced a recurrence, and the 5-year Disease Free Survival (DFS) was 90.0%. Compared to 2018 IB1 staged patients, new IB2 had significantly decreased 5-year DFS, 78.6% vs. 92.9%, *p* = 0.006 whereas IIIC patients had similar 5-year DFS (91.7%, *p* = 0.95). In the subgroup of patients with FIGO 2018 IB1 stage, the presence of LVSI was associated with a significant decrease in DFS (82.5% vs. 95.8%, *p* = 0.04). Conclusions: LVSI is associated with decreased 5-year DFS in IB1 2018 FIGO stage and LVSI status should be considered in early-stage cervical cancer for a more precise risk assessment.

## 1. Introduction

With nearly 570,000 new cases/year and 310,000 deaths/year, cervical cancer remains a major cause of morbidity and mortality from cancer, especially in developing countries where screening programs are not always available [1,2]. In addition to cervical screening, the incidence of cervical cancer will be decreased further with human papillomavirus vaccination [3].

Tailored treatments should be provided to patients according to an adequate staging which is usually based on preoperative clinical examination, pelvic MRI and PET-scans [1,4]. An optimal staging system may precisely reflect the extent of the disease and determine accurately prognostic groups of patients reflecting survival outcomes according to different predictive factors. Moreover, terminology may be harmonized as well as the comparison of patient outcomes from different centers. In the former 2014 FIGO (International Federation of Gynecology and Obstetrics) staging system, macroscopic early-stage cervical cancer (IB1 and IB2 2014 FIGO) was subdivided in two groups according to a cut-off at 4 cm (IB1 < 4 cm and IB2 > 4 cm, respectively). In addition, node status was not taken into account although nodal involvement has been widely described as a major prognostic factor [5,6,7,8] and five-year disease-free survival falls from 88% to 57% when lymph node metastasis is documented [9].

The purpose of modifying a tumor staging system is to incorporate already well-established prognostic factors, allowing one to stratify cases and also leading to tailored treatment approaches. It has been known for a long time that a large tumor size and the presence of lymph nodes metastasis are both linked with an increased recurrence rate and poorer survival among cervical cancer patients. In fact, these variables have been used to guide therapy, although they were not incorporated into the 2009 FIGO staging system. As previously for endometrial cancer [10] and ovarian cancer [11], FIGO classification of cervical cancer has been revised in 2018 [12]. Compared to previously, two major changes were provided: the addition of IIIC stage in case of node involvement and the revision of stage IB. Lymph node metastases diagnosed either on imaging (r) or pathology (p) have been now incorporated into a new IIIC FIGO stage and more specifically stage IIIC1 for pelvic lymph node metastasis only or stage IIIC2 for paraaortic lymph node metastasis. Stage IB are now subdivided in three sub-stages rather than two with respect to 2-cm cut-off in tumor size with the addition of a new IB3 stage: stage IB1 (<2 cm), stage IB2 disease (2–3.9 cm), and stage IB3 (≥4 cm).

Although lymphovascular space invasion (LVSI) has been described as an independent risk-factor of recurrence in early-stage cervical cancer [13,14] and defined intermediate and high-risk cervical cancer according to the ESGO guidelines [4], this factor remains controversial [15,16] and was not included in the last revised 2018 FIGO classification [12]. By contrast, the presence of LVSI has been integrated in the decision-making process in the last guidelines for endometrial cancer and indicates an intensification of adjuvant treatments [17].

The aim of this study was to assess the prognostic impact of LVSI in IB1 stage of the revised 2018 FIGO classification for cervical cancer.

## 2. Results

### 2.1. Population Characteristics

**Figure 1:**
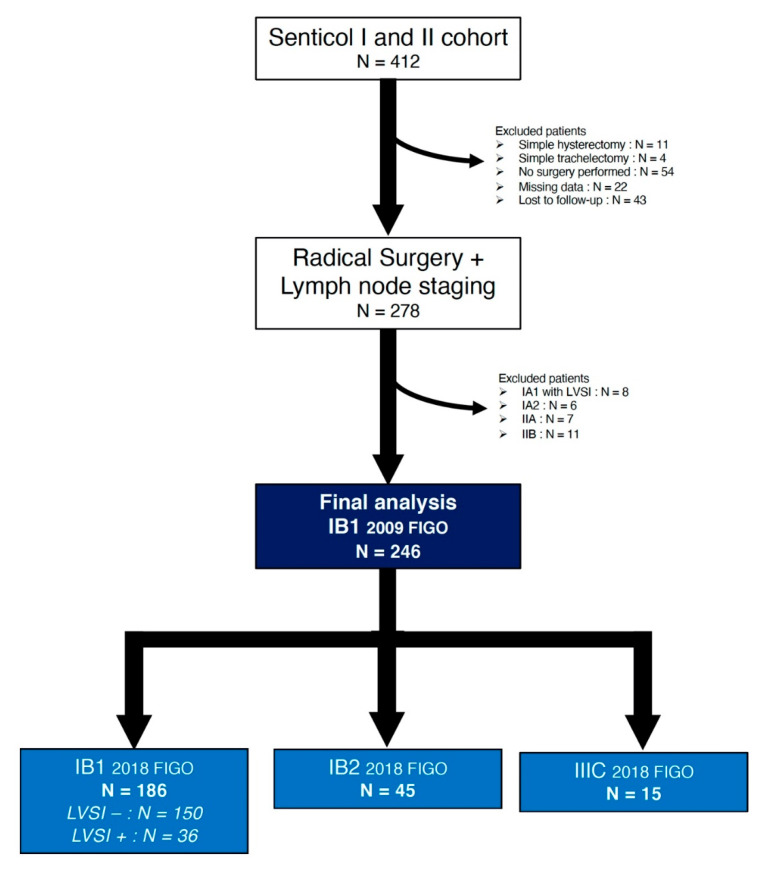
Flow-chart of the population study.

Among the 412 patients who were enrolled in both studies, 246 patients had IB1 disease stage according to 2009 FIGO classification and fulfilled the inclusion criteria (Figure 1).

The clinicopathological characteristics of the study population are presented in Table 1. The median age was 42 years (22–85) and the median BMI was 22.7 kg/m^2^ (14.6–41.4). The majority of patients had squamous cell carcinoma (166 patients, 67.5%). Most patients had a radical hysterectomy (195 patients, 79.3%) and surgical procedures were mainly performed with a minimally invasive approach (229 patients–93.1%). SLN biopsy was performed exclusively in 77 patients (31.3%) whereas pelvic lymphadenectomy in addition to SLN biopsy was performed in 169 patients (68.7%). At the final pathologic examination, the tumor size was larger than 20 mm in 46 patients (18.7%). Surgical margins were positive in nine patients (3.7%). Fifteen patients (6.1%) had at least one metastatic lymph node. In most of cases, adjuvant treatment was not required (186 patients, 75.6%).

### 2.2. FIGO Stage Modification

The entire cohort was staged as pIB1 according to the 2009 FIGO classification. Modification of the FIGO classification for cervical cancer resulted in an upstaging change for 60 patients (24.4%): 45 patients (18.3%) to IB2 due to tumor size and 15 patients (6.1%) to IIIC due to positive nodes.

Fifteen patients had at least one metastatic node: macrometastases were found in seven patients (2.8%) and micrometastases in eight patients (3.3%). Among these 15 patients, four patients had tumor size larger than 20 mm. Moreover, nine patients (3.7%) had ITCs and were nonetheless upstaged as IB2 in five cases and remained staged as IB1 in four cases in the new classification. Among these 15 patients, the median number positive node was one per patient [1,2,3] and only one node was positive in nine patients, corresponding in all cases to one SLN, except for one patient. Most of these patients had pelvic positive nodes only (14 patients) (IIIC1) whereas only one patient had also para-aortic positive nodes (IIIC2).

To assess the prognostic impact of LVSI in the subpopulation of IB1 patients, the 186 patients staged as IB1 in the new classification were subdivided in two groups: 150 patients without LVSI (IB1 LVSI−) and 36 patients with LVSI (IB1 LVSI+) (Table 2). Between the two groups, there were no differences in terms of age, histology and type of surgical procedure. Compared to IB1 LVSI−, IB1 LVSI+ patients were more likely to have a bigger tumor (7.5 ± 7.4 mm versus 2.9 ± 5.2 mm, *p* < 0.0001) and deeper stromal invasion (5.4 ± 6.1 mm versus 2.1 ± 4.9 mm, *p* = 0.001). Patients IB1 with LVSI underwent more frequent postoperative brachytherapy (25% versus 6.7%) and external beam radiotherapy (EBRT) (8.3% versus 0.7%), *p* = 0.0002.

### 2.3. Survical Outcomes

The median duration follow-up was 48 months (4–127). For the whole population, the 5 year-DFS and 5 year-DSS were 90% (Standard error: 2.3%) and 96.2% (Standard error: 1.3%), respectively.

**Figure 2:**
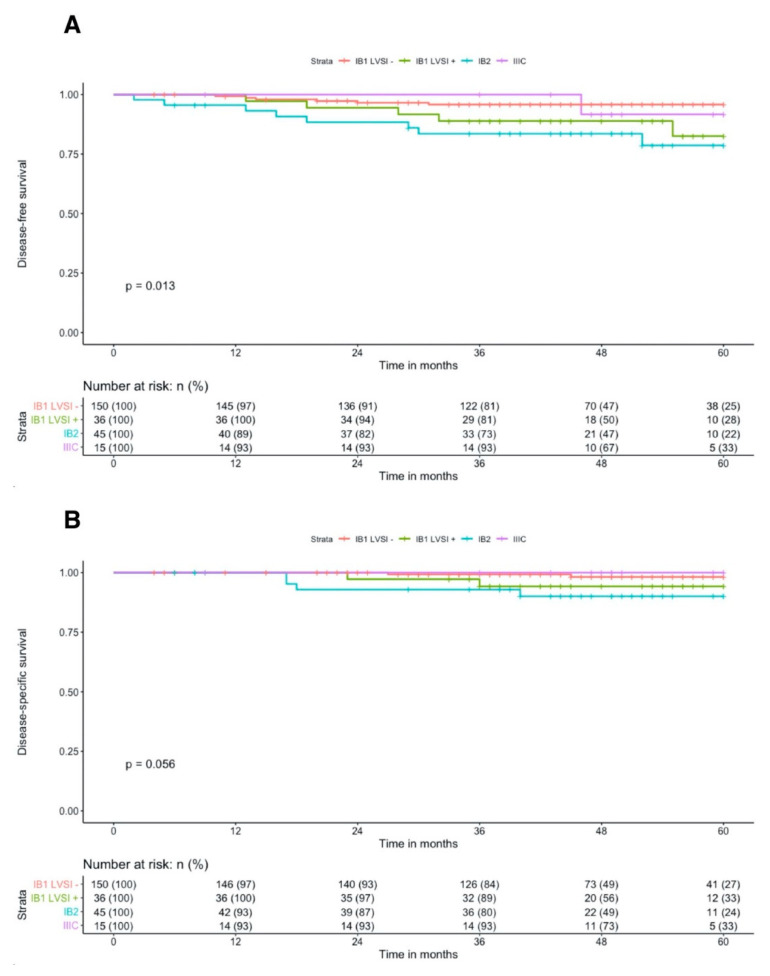
Kaplan-Meier 5-year disease-free survival (**A**) and 5-year disease-specific survival (**B**) curves according to 2018 FIGO classification.

During the follow-up, 20 patients experienced a recurrence (8.1%): four vaginal only, three nodal only, four pelvic only, two with nodal and vaginal, and seven distant metastases. Compared to newly IB1 staged patients, new IB2 had significantly decreased 5-year DFS, 78.6% vs. 92.9%, *p* = 0.006 whereas IIIC patients had similar 5-year DFS (91.7%, *p* = 0.95) (Figure 2A). In the subgroup of patients with FIGO 2018 IB1 stage, the presence of LVSI was associated with a three-fold increased risk of cervical cancer recurrence (HR = 3.33, 95%IC = [1.02–10.91], *p* = 0.047) (Table 3) and a significant decrease in 5-year DFS (82.5% vs. 95.8%, *p* = 0.04). After adjusting for tumor size and depth of stromal invasion, the presence of LVSI was independently associated with cervical cancer recurrence (HR = 3.99, 95%IC = [1.05–15.24], *p* = 0.043).

Eight patients (3.2%) died from cervical cancer. By applying the revised 2018 FIGO classification, IB2 patients had significant decreased 5-year DSS compared to IB1, 90.0% versus 97.3%, *p* = 0.02 (Figure 2B). Among IIIC patients, no death was reported. The cox regression analysis revealed that IB1 LVSI+ tended to be associated with an increased risk of cervical cancer mortality without reaching a statistically significant set (HR = 3.99, 95%IC = (0.56–28.34), *p* = 0.17) (Table 4).

## 3. Discussion

The goal of the present study was to determine whether LVSI had an impact on the prognosis of IB1 patients according to 2018 FIGO classification through two French prospective multicentric cohorts. The clinical impact of LVSI in early-stage cervical cancer is still subject to debate. Some studies highlighted that LVSI was an independent risk-factor in early-stage cervical cancer [13,14,18], whereas others supported that LVSI alone was not a prognostic factor [15,16]. The first prospective study conducted by the Gynecologic Oncology Group (GOG-49) on 645 patients with FIGO stage IB squamous cell carcinoma of the cervix have shown that 3-year disease-free survival was significantly lower in LVSI positive patients (77.0% vs. 88.9%, *p* = 0.0001) and was independently influenced by tumor size, stromal invasion depth, and the presence of LVSI (*p* = 0.006) [19]. We emphasized that the presence of LVSI had a clinical impact among newly staged IB1 patients on 5-year DFS but not on 5-year DSS. The LVSI status is taken into account in most international guidelines for treatment algorithm [1,4,20], but this risk-factor was nonetheless not included in the last revised 2018 FIGO classification. One of the main reasons is the lack of consensus to diagnose LVSI in tissue samples due to an important intra- and interobserver variability [1,13].

In this cohort, larger tumor size and deeper stromal invasion were associated with the presence of LVSI among 2018 IB1 patients. Two other studies underlined that the depth of stromal invasion was a predictive factor of the presence of LVSI [14,21]. We did not find any association between the histologic type and the presence of LVSI, although some studies put in evidence that squamous cell carcinomas were more often associated with LVSI than adenocarcinomas or adenosquamous carcinomas [18,21]. This point raises the question of the impact of histologic type on survival outcomes which still remains controversial [22]. Some authors reported worse outcomes of patients with adenocarcinomas, as compared to squamous cell carcinomas [23] which would be linked to a higher incidence of pelvic lymph node involvement. However, it seems more likely that worse outcomes are related to an ineffective adjuvant treatment, rather than a potentially higher incidence of lymph node involvement in adenocarcinomas [24].

We highlighted that IB1 LVSI+ patients had significantly more brachytherapy and EBRT (*p* = 0.0002). Yan et al. also reported that the incidence of LVSI was significantly associated with treatment after surgery (*p* < 0.001) [14]. The authors explained these results by the association between the LVSI and other high-risk factors such as higher FIGO stage and lymph node metastases. The same results were reported by Weyl et al. who have shown that patients with LVSI received more brachytherapy (*p* = 0.008) and more external radiotherapy (*p* < 0.0001) than patients without it [21]. The 5-year DFS and 5-year overall survival were similar between the group with LVSI and the group without because of the different adjuvant treatments received. By contrast, we found a significant decrease in 5-year DFS in IB1 LVSI+ patients compared to IB1 LVSI− (82.5% vs. 95.8%, *p* = 0.04). IB1 LVSI+ patients received adjuvant EBRT in only 8.3% of cases, whereas in the study by Weyl et al., patients with LVSI received neoadjuvant EBRT in 25.0% of cases and adjuvant EBRT in 27.8% [21]. We believe that that difference may be explained by the fact that our analysis was strictly limited to the subgroup of 2018 IB1 patients and did not include IB2 and IIIC patients and therefore EBRT was less indicated.

Tumor size is a well-known major prognostic factor in cervical cancer which is mainly correlated with nodal involvement and worst oncologic outcomes [25,26,27]. The Cox analysis revealed that new IB2 was associated with a significant increased risk of recurrence (HR = 3.31, 95%IC = [1.33–8.23], *p* = 0.003) and death (HR = 4.35, 95%IC = [1.09–17.4], *p* = 0.04). The subdivision of former 2009 IB1 into two substage according to a 2-cm cut-off enhanced to identify the subgroup of patients with a distinct prognosis in terms of 5-year DFS. Stepwising by 2-cm increments in tumor size in early-stage cervical cancer is correlated to current clinical practice. On one hand, patients should have an IB1 tumor smaller than 2 cm with no evidence of lymph node involvement to be eligible for radical trachelectomy in the scope of fertility-sparing management [4,28]. On the other hand, since the publication of the LACC trial results which highlighted the recurrence risk associated with minimally invasive surgery in cervical cancer, it has been suggested that a specific subgroup of patients with a small IB1 less than 2 cm may still benefit from this kind of approach without compromising survival [29,30]. Matsuo et al. reported 5-year DSS rates of 97.0%, 92.1%, and 83.1% for women with 2018 FIGO stages IB1, IB2, and IB3, respectively, *p* < 0.001. In our cohort, 5-year DSS was 97.3% and 90.0% for IB1 and IB2 patients respectively (*p* = 0.04).

We have shown that the upstaging modification was only 24.4%. De Gregorio et al. and Ayhan et al. reported an upstaging modification of 55% and 87.5%, including 32.1% and 35.3% due to lymph nodes positivity, respectively [31,32]. Lymph node metastases are recognized as a major prognosis factor in cervical cancer, justifying its inclusion in the revised FIGO classification. In our cohort, the rate of node-positive patients was fairly low (6.1%). This low rate might be explained by the selective exclusion criteria of SENTICOL 1 and 2 which excluded locally advanced cervical cancer and suspicious nodes at preoperative imaging. These exclusion criteria also resulted in the absence of patients with IB3 stage disease and might induce selection bias. Among the 15 patients with positive nodes, only 1 had para-aortic involved nodes (IIIC2), and therefore distinguishing IIIC1 and IIIC2 was not appropriate. In our study, the 5-year DFS of IIIC patients was 91.7% and was higher than that reported previously by Ayhan et al. of 75.2% [32]. Matsuo et al. hypothesized that IIIC1 disease stage may correspond to a heterogenous group of patients who may have different prognoses based on different tumor characteristics such as local tumor burden and tumor size [33]. The authors highlighted that, among patients with IIIC1 stage disease, 5-year DSS was correlated with tumor size and decreased significantly at 74.8%, 58.7% and 39.3% for T1, T2 and T3 stage, respectively. These results were confirmed by McComas et al. who did not find any statistically significant differences in 5-year DSS in IIIC1 patients and local T stage 1A, 1B1, and 1B2 disease [34]. In this study, only 4 of 15 node-positive patients had tumor size larger than 20 mm. Furthermore, the clinical impact of low-volume metastases remained controversial. Cibula et al. have shown in a retrospective analysis that micrometastasis had a worse impact on overall survival, similar to that of macrometastasis [35], whereas Guani et al. highlighted, in a smaller but prospective cohort, that low volume metastases might have no impact on 3-year PFS in early-stage cervical cancer [36]. In our cohort, 7 patients of 15 node-positive patients had micrometastases. The survival at IIIC1 disease stage may vary according to the size of lymph node involved, bulky macrometastasis versus micrometastasis and ITCs [33].

One of the main limitations of this study is the retrospective analysis of two databases which were not designed to our objectives. However, clinical, surgical and pathologic data were prospectively recorded in a quality-check database. Another pitfall is the type of surgical approach. In this cohort, most of patients underwent minimally invasive surgery which was the gold standard during the inclusion period of both studies. It has been demonstrated that minimally invasive approach increased the risk of recurrence [29,37,38] and we believe that it might influence our results. To our knowledge, this study is one of the first analyses that argue in favor of the inclusion of LVSI status to more precisely discriminate IB1 2018 FIGO patients and contributes to the validation of the revised 2018 FIGO staging system for cervical cancer. We support the idea that IB1 2018 FIGO stage might be divided in two substages according to the LVSI status.

## 4. Materials and Methods

### 4.1. Patient Selection

A secondary analysis of two prospective multicentric trials on Sentinel Lymph Node (SLN) biopsy for cervical cancer was performed. The designs of both studies have been described elsewhere [39,40]. Briefly, in SENTICOL I, patients were included between 2005 and 2007 from 7 French gynecological oncology centers and, in SENTICOL II, between 2009 and 2012 from 23 French gynecological oncology centers. In both studies, all patients had early-stage cervical cancer up to IIA1 FIGO 2009 classification, no suspicious nodes at preoperative imaging and underwent SLN mapping.

In the present study, we included patients with 2009 pFIGO IB1 stage, undergoing radical surgery and lymph node staging. The exclusion criteria were patients with IA1 with emboli and IA2 2009 FIGO stage, patients with IIA and IIB 2009 FIGO stage, patients with no lymph node staging and patients who did not undergo radical surgery. According to the new 2018 FIGO staging system, the stage was defined retrospectively based on final pathologic examination.

This study was approved by institutional review board of the Paris Descartes (HEGP-Broussais) (Ethical code: DRRC AOR 03063) and Lyon’s civil Hospices’ Ethical Committee (Ethical code: 2008-A01369-46). An informed consent form allowing the use of data for secondary analyses was signed by the patients included.

### 4.2. Data Analysis

From both databases, patient demographics, tumor characteristics, treatment type, and follow-up data were extracted and analyzed. Pathologic reports were reviewed and the FIGO stage was modified according to the 2018 FIGO classification. To assess the impact of LVSI, patients newly staged as IB1 in the 2018 FIGO classification were subdivided according to the presence or lack thereof of LVSI. The presence of LVSI was defined by the presence of tumor cells in the lumen of vessels or lymphatic channels.

All patients underwent radical surgery consisting of radical hysterectomy type B or type C from the Querleu-Morrrow classification or in radical trachelectomy. In Senticol 1, patients underwent a systematic pelvic lymphadenectomy after SLN biopsy. In SENTICOL II, patients underwent a SLN biopsy and an additional pelvic lymphadenectomy was performed according to the randomization group. In the case of no SLNs detected on one-side, an homolateral hemipelvic lymphadenectomy was performed. Node-negative patients were defined if bilateral SLN was free of disease or all non-SLNs were negative. Isolated tumor cells (ITCs) were defined as <0.2 mm, micrometastases as between 0.2 and 2 mm, and macrometastases as >2 mm [41]. Node-positive patients were defined if at least one node was positive for micrometastases or macrometastases (SLNs and/or non-SLNs) whereas patients with ITCs were considered as node-negative patients [42]. Tumor size was macroscopically measured in surgical specimens.

Adjuvant treatment was decided at the discretion of each multidisciplinary team in case of positive surgical margins, positive nodes, tumor larger than 20 mm or positive LVSI.

### 4.3. Statistical Analysis

Categorical variables were expressed as *n* (%) and were compared by applying chi-square test. Continuous variables were expressed as mean [range] and were compared by applying the Student’s t-test. Disease-free survival (DFS) was defined as the interval in months between the date of surgery and the date of first recurrence or the date of last follow-up for patients who were still alive without any recurrence. Disease-specific survival (DSS) was defined as the interval in months between the date of surgery and the date of death from the disease or the date of last visit. Five-year DFS and 5-year DSS curves were built using Kaplan-Meier method, and the log-rank test was used for survival comparisons. To assess the impact of FIGO stage on 5 year-DFS and 5 year-DSS, a Cox proportional hazards regression model was applied to obtain hazard ratios (HRs) and a 95% confidence interval (CI). All statistical tests were two-sided and *p* values lower than 0.05 were retained as a significance set. All statistical tests were performed using XLStat Biomed software (AddInsoft, Paris, France) and R studio (Version 1.2.5042).

## 5. Conclusions

The revised 2018 FIGO classification for cervical cancer provides more accurate prognosis reflection by identifying the subpopulation at high-risk of recurrences among former IB1 2009 FIGO patients. LVSI is associated with decreased 5-year DFS in IB1 2018 FIGO stage and particular attention should be paid to LVSI status in early-stage cervical cancer for a more precise risk assessment.

## Figures and Tables

**Table 1:** Patient characteristics.

Variable	Total Population *N* = 246	
	*N* Patients or Median	% or (Range)
Age at diagnosis [years]		
All patients	42	(22–85)
<50	181	73.6
50–70	56	22.8
>70	9	3.7
Body Mass-Index [kg/m^2^]		
All patients	22.7	(14.6–41.4)
<18.5	20	8.1
18.5–25	154	62.6
<25–30	43	17.5
>30	29	11.8
Menopausal status		
Premenopausal	174	70.7
Postmenopausal	72	29.3
Histology		
Squamous cell carcinoma	166	67.5
Adenocarcinoma	74	30.1
Other type	6	2.4
Grade of differenciation		
G1	77	43.0
G2	65	36.3
G3	37	20.7
Not specified	65	-
Conisation		
Yes	150	61.0
No	96	39.0
Preoperative brachytherapy		
Yes	66	26.8
No	180	73.2
Surgical procedure		
Type of surgery		
Radical Hysterectomy	199	80.9
Radical Trachelectomy	47	19.1
Type of surgical approach		
Minimal Invasive Surgery	229	93.1
Laparotomy	17	6.9
Type of Lymph node staging		
SLN alone	77	31.3
SLN + Pelvic lymphadenectomy	169	68.7
Final pathologic examination		
Tumor size		
<20 mm	200	81.3
≥20 mm	46	18.7
Deep stromal invasion		
<10 mm	178	80.2
≥10 mm	44	19.8
Not specified	24	-
LVSI		
Yes	57	23.2
No	189	76.8
Positive margin		
Yes	9	3.7
No	237	96.3
Patients with ≥1 positive node		
Yes	15	6.1
No	231	93.9
Adjuvant treatment		
None	186	75.6
Brachytherapy	29	11.8
EBRT	11	4.5
CCR	20	8.1
Outcomes		
Recurrence		
None	226	91.5
Nodal	5	2.0
Vaginal	5	2.0
Pelvic	5	2.0
Distant metastases	6	2.4
Status		
Alive	238	96.8
Dead	8	3.2

**Table 2:** Characteristics of IB1 2018 International Federation of Gynecology and Obstetrics (FIGO) patients.

Predictive Variable	IB1 LVSI− *N* = 150		IB1 LVSI+ *N* = 36		*p*
	*N* Patients or Mean ± SD	% or (Range)	*N* Patients or Mean ± SD	% or (Range)	
Age (years)					
All patients	43.8 ± 12.3	(22–85)	43.1 ± 12.3	(26–72)	0.74
<50	112	74.7	25	69.4	0.68
50–70	32	21.3	10	27.8	
> 70	6	4.0	1	2.8	
BMI [kg/m^2^]					
All patients	23.9 ± 5.2	(14.6–41.4)	23.5 ± 4.8	(17.4–37.7)	0.72
<18.5	10	6.7	1	2.8	0.75
18.5–25	97	64.7	26	72.2	
<25–30	26	17.3	5	13.9	
>30	17	11.3	4	11.1	
Menopausal status					
Premenopausal	107	71.3	25	69.4	0.83
Postmenopausal	43	28.7	11	30.6	
Histology					
Squamous cell carcinoma	101	67.3	25	69.4	0.43
Adenocarcinoma	46	30.7	9	25.0	
Other type	3	2.0	2	5.6	
Grade of differenciation					
G1	47	45.2	10	35.7	0.22
G2	39	37.5	9	32.1	
G3	18	17.3	9	32.1	
Not specified	46	-	8	-	
Conisation					
Yes	100	67.1	22	61.1	0.49
No	49	32.9	14	38.9	
Preoperative brachytherapy					
Yes	47	31.3	7	19.4	0.16
No	103	68.7	29	80.6	
Surgical procedure					
Type of surgery					
Radical Hysterectomy	118	78.7	30	83.3	0.53
Radical Trachelectomy	32	21.3	6	16.7	
Type of surgical approach					
Minimal Invasive Surgery	143	95.3	35	97.2	0.62
Laparotomy	7	4.7	1	2.8	
Type of Lymph node staging					
SLN alone	58	38.7	9	25.0	0.12
SLN + Pelvic lymphadenectomy	92	61.3	27	75.0	
Final pathologic exam					
Tumor size (mm)	2.9 ± 5.2	(0–18)	7.5 ± 7.4	(0–18)	**<0.0001**
Deep stromal invasion					
Mean	2.1 ± 4.9	(0–20)	5.4 ± 6.1	(0–20)	**0.001**
<10 mm	132	94.3	23	67.6	**<0.0001**
≥10 mm	8	5.7	11	32.4	
Not specified	10	-	2	-	
Positive margin					
Yes	5	3.3	2	5.6	0.53
No	145	96.7	34	94.4	
Presence of ITCs					
Yes	3	2.0	1	2.8	0.77
No	147	98.0	35	97.2	
Adjuvant treatment					
None	136	90.7	24	66.7	**0.0002**
Brachytherapy	10	6.7	9	25.0	
EBRT	1	0.7	3	8.3	
CCR	3	2.0	0	0.0	

EBRT: External Beam Radiotherapy; CCR: chemoradiotherapy. In bold: statistically significant.

**Table 3:** Cox proportional Hazards models of 5-year Disease-free survival according to the 2009 FIGO and the revised 2018 FIGO staging systems.

2009 FIGO Classification				2018 FIGO Classification							2018 FIGO Classification with LVSI Status						
2009 FIGO	*n*	Number of events	5-year DFS	2018 FIGO	*n*	Number of events	5-year DFS	HR	95% CI	*p*	2018 FIGO	*n*	Number of events	5-year DFS	HR	95% CI	*p*
IB1	246	20	90.0%	IB1	186	11	92.9%	1			IB1 LVSI−	150	6	95.8%	1		
											IB1 LVSI+	36	5	82.5%	3.33	1.02–10.91	***0.047***
				IB2	45	8	78.6%	3.31	1.33–8.23	**0.003**	IB2	45	8	78.6%	4.85	1.68–13.98	***0.003***
				IIIC	15	1	91.7%	1.09	0.14–8.44	0.93	IIIC	15	1	91.7%	1.60	0.19–13.27	*0.66*

In bold: statistically significant.

**Table 4:** Cox proportional Hazards models of 5-year Disease-specific survival according to the 2009 FIGO and the revised 2018 FIGO staging systems.

2009 FIGO Classification				2018 FIGO Classification							2018 FIGO Classification with LVSI Status						
2009 FIGO	*n*	Number of events	5-year DFS	2018 FIGO	*n*	Number of events	5-year DFS	HR	95% CI	*p*	2018 FIGO	*n*	Number of events	5-year DFS	HR	95% CI	*p*
IB1	246	8	96.2%	IB1	186	4	97.3%	1			IB1 LVSI−	150	2	98.2%	1		
											IB1 LVSI+	36	2	94.2%	3.99	0.56–28.34	0.17
				IB2	45	4	90%	4.35	1.09–17.4	**0.04**	IB2	45	4	90.0%	6.96	1.27–38.01	***0.03***
				IIIC	15	0	100%	NA	NA	NA	IIIC	15	0	100%	NA	NA	NA

In bold : statistically significant.
